# Supplementary material for: Molecular Characterization of Mycobacterium ulcerans DNA Gyrase and Identification of Mutations Reducing Susceptibility to Quinolones In Vitro
Source: Antimicrob Agents Chemother. 2022 Mar 28;66(4):e01902-21. doi: 10.1128/aac.01902-21 (PMC9017346; doi:10.1128/aac.01902-21)
Supplement: Supplemental file 1 — Fig. S1. Download aac.01902-21-s0001.pdf, PDF file, 0.9 MB [file aac.01902-21-s0001.pdf]

# Supplementary figure

## Molecular characterization of *Mycobacterium ulcerans* DNA gyrase and identification of mutations reduced susceptibility against quinolones *in vitro*

Hyun Kim,[a#] Shigtarou Mori,[a] Tsuyoshi Kenri,[a] Yasuhiko Suzuki[b,c]

[a]Department of Bacteriology II, National Institute of Infectious Diseases, Tokyo, Japan
[b]Division of Bioresources, Hokkaido University International Institute for Zoonosis Control, Sapporo, Japan
[c]International Collaboration Unit, Hokkaido University International Institute for Zoonosis Control

# Supplemental Figure Legend

**Suppl. Fig.**
**Multiple amino acid sequence alignment of Mycobacteria DNA GyrA subunit.**
The multiple alignment of Mycobacteria amino acid sequence was performed  by Genetyx ver.21 (https://www.genetyx.co.jp).
Amino acids sequence were showed the N-terminus of DNA GyrA subunit (1-500 amino acid) from Mycobacteria.
Same amino acid residues are indicated by asterisk. Red box indicated the Quinolone-resistance determining regions (QRDRs).

```
1_M.marinum                  ------------------------MQRSYIDYAMSVIVGRALPEVRDGLK 26
2_M.ulcerans_shinshuense     MTDTTLPPGGEASDRIEPVDIQQEMQRSYIDYAMSVIVGRALPEVRDGLK 50
3_M.ulcerans_Agy99           MTDTMLPPGGEASDRIEPVDIQQEMQRSYIDYAMSVIVGRALPEVRDGLK 50
4_M.thermoresistibile        MTDTTLPPGGEAGDRIEPVDIQQEMQRSYIDYAMSVIVGRALPEVRDGLK 50
5_M.tuberculosis             -MTDTTLPPDDSLDRIEPVDIEQEMQRSYIDYAMSVIVGRALPEVRDGLK 49
6_M.leprae                   MTDITLPPGDGSIQRVEPVDIQQEMQRSYIDYAMSVIVGRALPEVRDGLK 50
                                                     **************************
                                                                    91  95
1_M.marinum                  PVHRRVLYAMYDSGFRPDRSHAKSARSVAETMGNYHPHGDASIYDTLVRM 76
2_M.ulcerans_shinshuense     PVHRRVLYAMYDSGFRPDRSHAKSARSVAETMGNYHPHGDASIYDTLVRM 100
3_M.ulcerans_Agy99           PVHRRVLYAMYDSGFRPDHSHAKSARSVAETMGNYHPHGDASIYDTLVRM 100
4_M.thermoresistibile        PVHRRVLYAMYDSGFRPDRSHAKSARSVAETMGNYHPHGDASIYDTLVRM 100
5_M.tuberculosis             PVHRRVLYAMFDSGFRPDRSHAKSARSVAETMGNYHPHGDASIYDSLVRM 99
6_M.leprae                   PVHRRVLYAMLDSGFRPDRSHAKSARSVAETMGNYHPHGDASIYDTLVRM 100
                             ********** *******:**************************:****

1_M.marinum                  AQPWSLRYPLVDGQGNFGSPGNDPPAAMRYTEARLTPLAMEMLREIDEET 126
2_M.ulcerans_shinshuense     AQPWSLRYPLVDGQGNFGSPGNDPPAAMRYTEARLTPLAMEMLREIDEET 150
3_M.ulcerans_Agy99           AQPWSLRYPLVDGQGNFGSPGNDPPAAMRYTEARLTPLAMEMLREIDEET 150
4_M.thermoresistibile        AQPWSMRYPLVDGQGNFGSPGNDPPAAMRYTEARLTPLAMEMLREIDEET 150
5_M.tuberculosis             AQPWSLRYPLVDGQGNFGSPGNDPPAAMRYTEARLTPLAMEMLREIDEET 149
6_M.leprae                   AQPWSLRYPLVDGQGNFGSPGNDPPAAMRYCEARLTPLAMEMLREIDEET 150
                             *****:************************ *******************

1_M.marinum                  VDFIPNYDGRVQEPTVLPSRFPNLLANGSGGIAVGMATNIPPHNLRELAE 176
2_M.ulcerans_shinshuense     VDFIPNYDGRVQEPTVLPSRFPNLLANGSGGIAVGMATNIPPHNLRELAE 200
3_M.ulcerans_Agy99           VDFIPNYDGRVQEPTVLPSRFPNLLANGSGGIAVGMATNIPPHNLRELAE 200
4_M.thermoresistibile        VDFVPNYDGRVQEPTVLPSRFPNLLANGSGGIAVGMATNIPPHNLRELAE 200
5_M.tuberculosis             VDFIPNYDGRVQEPTVLPSRFPNLLANGSGGIAVGMATNIPPHNLRELAD 199
6_M.leprae                   VDFISNYDGRVQEPMVLPSRFPNLLANGSGGIAVGMATNIPPHNLYELAD 200
                             ***:.********* ****************************** ***:

1_M.marinum                  AVFWCLENHDADEEATLAAVTERVKGPDFPTSGLIVGTQGISDAYKTGRG 226
2_M.ulcerans_shinshuense     AVFWCLENHDADEEATLAAVTDRVKGPDFPTSGLIVGTQGISDAYKTGRG 250
3_M.ulcerans_Agy99           AVFWCLENHDADEEATLAAVTDRVKGPDFPTSGLIVGTQGISDAYKTGRG 250
4_M.thermoresistibile        AVYWCLENYEADEETTLAAVMERVKGPDFPTAGLIVGSQGIHDAYTTGRG 250
5_M.tuberculosis             AVFWALENHDADEEETLAAVMGRVKGPDFPTAGLIVGSQGTADAYKTGRG 249
6_M.leprae                   AVFWCLENHDADEETMLVAVMERVKGPDFPTAGLIVGSQGIADAYKTGRG 250
                             **:*.***::****  *.**  *********:*****:**  ***.****

1_M.marinum                  SIRMRGVVEIEEDSRGRTSLVITELPYQVNHDNFITSIAEQVRDGKLAGI 276
2_M.ulcerans_shinshuense     SIRMRGVVEIEEDSRGRTSLVITELPYQVNHDNFITSIAEQVRDGKLAGI 300
3_M.ulcerans_Agy99           SIRMRGVVEIEEDSRGRTSLVITELPYQVNHDNFITSIAEQVRDGKLAGI 300
4_M.thermoresistibile        SIKMRGVVDIEEDSRGRTSLVITELPYQVNHDNFIASIAEQVRDGKIAGI 300
5_M.tuberculosis             SIRMRGVVEVEEDSRGRTSLVITELPYQVNHDNFITSIAEQVRDGKLAGI 299
6_M.leprae                   SIRIRGVVEVEEDSRGRTSLVITELPYQVNHDNFITSIAEQVRTGRLAGI 300
                             **::****::*************************:******* *::***

1_M.marinum                  SNIEDQSSDRVGLRIVVEIKRDAVAKVVLNNLYKHTQLQTSFGANMLSIV 326
2_M.ulcerans_shinshuense     SNIEDQSSDRVGLRIVVEIKRDAVAKVVLNNLYKHTQLQTSFGANMLSIV 350
3_M.ulcerans_Agy99           SNIEDQSSDRVGLRIVVEIKRDAVAKVVLNNLYKHTQLQTSFGANMLSIV 350
4_M.thermoresistibile        ANVEDQSSDRVGLRMVIELKRDAVAKVVLNNLYKHTQLQTSFGVNMLAIV 350
5_M.tuberculosis             SNIEDQSSDRVGLRIVIEIKRDAVAKVVINNLYKHTQLQTSFGANMLAIV 349
6_M.leprae                   SNVEDQGSDRVGVRIVIEIKRDAVAKVVLNNLYKHTQLQTSFGANMLSIV 350
                             :*:***.*****:*:*:*:*********:**************.***:**

1_M.marinum                  DGVPRTLRLDQMIRYYVEHQLDVIIRRTTYRLRKANERAHILRGLVKALD 376
2_M.ulcerans_shinshuense     DGVPRTLRLDQMIRYYVEHQLDVIIRRTTYRLRKANERAHILRGLVKALD 400
3_M.ulcerans_Agy99           DGVPRTLRLDQMIRYYVEHQLDVTIRRTTYRLRKANERAHILRGLVKALD 400
4_M.thermoresistibile        DGVPRTLRLDQMIRLYTDHQIDVIVRRTRYRLRKANERAHILRGLVKALD 400
5_M.tuberculosis             DGVPRTLRLDQLIRYYVDHQLDVIVRRTTYRLRKANERAHILRGLVKALD 399
6_M.leprae                   DGVPRTLRLDQMICYYVEHQLDVIVRRTTYRLRKANERAHILRGLVKALD 400
                             ***********:*  *.:**:** :*** *********************

1_M.marinum                  ALDEVIALIRASQTVDIARAGLIELLDIDEIQAQAILDMQLRRLAALERQ 426
2_M.ulcerans_shinshuense     ALDEVIALIRASQTVDIARAGLIELLGIDEIQAQAILDMQLRRLAALERQ 450
3_M.ulcerans_Agy99           ALDEVIALIRASQTVDIARAGLIELLGIDEIQAQAILDMQLRRLAALERE 450
4_M.thermoresistibile        ALDEVIALIRASETVDIARDGLMELLEIDEIQAQAILDMQLRRLAALERQ 450
5_M.tuberculosis             ALDEVIALIRASETVDIARAGLIELLDIDEIQAQAILDMQLRRLAALERQ 449
6_M.leprae                   ALDEVITLIRASQTVDIARVGVVELLDIDDIQAQAILDMQLRRLAALERQ 450
                             ******:*****:****** *.:*** **:*******************:

1_M.marinum                  RIVDDLAKIEAEIADLEDILAKPERQRAIVRDELAEIVDKHGDDRRTRII 476
2_M.ulcerans_shinshuense     RIVDDLAKIEAEIADLEDILAKPERQRAIVRDELAEIVDKHGDDRRTRII 500
3_M.ulcerans_Agy99           RIVDDLAKIEAEIADLEDILAKPERQRAIVRDELAEIVDKHGDDRRTRII 500
4_M.thermoresistibile        RIVDDLAKIEAEIADLEDILAKPERQRGIVRDELKEIVDKHGDDRRSRLI 500
5_M.tuberculosis             RIIDDLAKIEAEIADLEDILAKPERQRGIVRDELAEIVDRHGDDRRTRII 499
6_M.leprae                   RIIDDLAKIEVEIADLGDILAKPERRRGIIRNELTEIAEKYGDDRRTRII 500
                             **:*******.***** ********:*.*:*:** **.:::****:*:*
```
